# RETRACTION: The Effects of Different Regenerative Technologies and Materials on Wound Healing after Surgical Endodontic Therapy: A Meta‐Analysis

**DOI:** 10.1111/iwj.70309

**Published:** 2025-03-06

## Abstract

**RETRACTION:** WangG. , YuanF. , YingW. , and XuJ. , “The Effects of Different Regenerative Technologies and Materials on Wound Healing after Surgical Endodontic Therapy: A Meta‐Analysis,” International Wound Journal 20, no. 10 (2023): 4340–4348, 10.1111/iwj.14293.37437962 PMC10681480

The above article, published online on 12 July 2023, in Wiley Online Library (http://onlinelibrary.wiley.com/), has been retracted by agreement between the journal Editor in Chief, Professor Keith Harding; and John Wiley & Sons Ltd. Following an investigation by the publisher, all parties have concluded that this article was accepted solely on the basis of a compromised peer review process. The editors have therefore decided to retract the article. The authors did not respond to our notice regarding the retraction.

**RETRACTION:** G. Wang , F. Yuan , W. Ying , and J. Xu , “The Effects of Different Regenerative Technologies and Materials on Wound Healing after Surgical Endodontic Therapy: A Meta‐Analysis,” International Wound Journal 20, no. 10 (2023): 4340–4348, 10.1111/iwj.14293.37437962 PMC10681480

The above article, published online on 12 July 2023, in Wiley Online Library (http://onlinelibrary.wiley.com/), has been retracted by agreement between the journal Editor in Chief, Professor Keith Harding; and John Wiley & Sons Ltd. Following an investigation by the publisher, all parties have concluded that this article was accepted solely on the basis of a compromised peer review process. The editors have therefore decided to retract the article. The authors did not respond to our notice regarding the retraction.

